# An acute gastroenteritis outbreak associated with person-to-person transmission in a primary school in Shanghai: first report of a GI.5 norovirus outbreak in China

**DOI:** 10.1186/s12879-018-3224-4

**Published:** 2018-07-09

**Authors:** Jian Li, Xia Gao, Yu-Long Ye, Tang Wan, Hao Zang, Ping-Hua Mo, Can-Lei Song

**Affiliations:** 1 Clinical Research Center, Ruijin Hospital, Shanghai Jiao Tong University School of Medicine, 197 Ruijin 2nd Road, Shanghai, 200025 China; 2 Department of Acute Infectious Diseases Control, Jinshan District Center for Diseases Control and Prevention, 94 Weisheng Rd, Jinshan District, Shanghai, 201599 China; 3 Department of Microbiology, Jinshan District Center for Diseases Control and Prevention, Shanghai, China

**Keywords:** Gastroenteritis, GI.5, Norovirus, Outbreak

## Abstract

**Background:**

GII noroviruses are a common cause of acute gastroenteritis (AGE) outbreaks in institutional settings globally. However, AGE outbreaks caused by GI norovirus, especially the GI.5 genotype, are relatively uncommon.

**Methods:**

In February 2017, an AGE outbreak occurred in a primary school in Shanghai, China. An outbreak investigation was undertaken, and fecal specimens, rectal swabs, and environmental swabs were collected. Pathogen detection was performed and the positive specimens were characterized by gene sequencing.

**Results:**

The descriptive epidemiological analysis suggested that this outbreak, involving 19 cases in two classes (designated classes A and B), was a small-scale propagated epidemic and person-to-person transmission was the most plausible transmission mode. The outbreak comprised two peaks, with 15 cases occurring in class A during the main peak and four cases occurring in class B in the subsequent minor peak. The primary attack rate was 38% and the secondary attack rate was 10%. Univariable logistic regression indicated that contacting a suspect case was a risk factor for norovirus infection, with an unadjusted OR of 5.6 (95% CI: 1.6–20.1). Six fecal specimens were positive for GI norovirus, with a single genotype, GI.5 norovirus, being involved, as characterized by genotyping. This outbreak was the first reported outbreak of GI.5 norovirus in China.

**Conclusions:**

This study implies that GI.5 norovirus is a potential agent of outbreaks spread by person-to-person transmission in institutional settings. The investigation highlights the importance of sensitive surveillance, timely isolation of individuals who are ill, adequate hand hygiene, and proper environmental disinfection for prevention and control of AGE outbreaks caused by norovirus.

## Background

Noroviruses are recognized as the leading etiological agent of acute gastroenteritis (AGE) outbreaks in institutional settings, including schools, cruise ships, kindergartens, and health care facilities around the world [1–3], and there has been increasing concern about noroviruses over the past few years [4]. Humans are the sole known reservoir of norovirus infection, with a low infectious dose of < 10–100 virions [3]. People with AGE caused by norovirus usually have a short incubation period (24–48 h), vomiting, watery diarrhea, abundant viral shedding, and lack of durable immunity after infection. Noroviruses have characteristics of environmental persistence [5]. Moreover, asymptomatic norovirus carriers (who can shed the virus) pose a potential challenge to outbreak control [6]. Noroviruses can be classified into seven genogroups (GI to GVII) according to the amino acid sequence of the VP1 protein, and they can be divided into over 30 genotypes [7]. GII norovirus is most commonly associated with norovirus infections globally [8]. The GII.4 genotype is recognized as the major cause of AGE outbreaks due to the emergence of new variants every 2–4 years, induced by the evolutionary mechanisms of recombination and mutations [8]. The predominance of the GII.4 genotype in AGE outbreaks was shattered recently by a newly emergent GII.17 genotype, which became predominant in several countries in 2015, replacing the previously dominant GII.4 genotype [9]. In China, more AGE outbreaks caused by norovirus have been reported in recent years and they have resulted in a significant disease burden [10]. Most of the outbreaks in China were caused by GII norovirus [11, 12]. However, AGE outbreaks caused by GI norovirus were relatively uncommon. This paper describes an investigation of an AGE outbreak associated with person-to-person transmission in a primary school in Jinshan district, Shanghai, China, and the identification of GI.5 norovirus, which is the first reported outbreak by GI.5 norovirus infection in China.

## Methods

### Epidemiological investigations

On February 18, 2017, Jinshan District Center for Disease Control and Prevention was notified of an AGE outbreak in a primary school which started 2 days earlier (February 16). A group of epidemiologists were immediately convened to conduct an outbreak investigation to identify the source of the outbreak and mode of transmission and to implement control measures to manage the outbreak. The school is a state-owned primary school that has 1127 students in 30 classes (involving 5 grades) and 100 teachers and other staff. Suspect cases were defined as those who attended school and experienced measurable symptoms involving at least vomiting and/or diarrhea (three or more loose or watery stool in 24 h), and a date of onset from February 13, 2017. The confirmed cases involved suspect cases whose vomitus, stool specimens, or rectal swabs were positive for norovirus, as detected by reverse transcription-polymerase chain reaction (RT-PCR). We searched for suspect cases in the school and local health facilities. Each of the suspect cases was interviewed using a questionnaire to gather data on demographic characteristics, epidemiology, signs and symptoms, food and drinking water, and sanitation practices.

### Environmental investigations

We conducted an environmental investigation of the canteen and school, along with inspecting the food processing procedures and interviewing the food handlers about food hygiene. The sanitation of the drinking water supply was also investigated. No food samples were submitted as none remained from the outbreak.

### Microbiological investigations

As the vomitus had been immediately cleaned up by a teacher using a dry absorbent towel containing highly efficient peroxyacetic acid, we could not collect vomitus specimens. The teacher was wearing a mask and gloves when she cleaned up the vomitus. Stool specimens were submitted by six student cases (including the index case) and rectal swabs were collected from three members of staff who prepared food. Environmental swabs were also taken, including five swabs from canteen tableware, 2 swabs from the doorknobs of the classroom of the index case, and 2 swabs from the water outlet of a water dispenser. All the specimens were tested for enteropathogenic bacteria including *Salmonella* (GB4789.4–2016), *Escherichia coli* (GB4789.38–2012), and *Vibrio parahemolyticus* (GB4789.7–2013) using standard bacteria culture methods. All the specimens were also screened for enteropathogenic viruses including norovirus, rotavirus, adenovirus, and astrovirus. Viral RNA/DNA was extracted using a QIAamp® Viral RNA Mini Kit (QIAGEN, Germany) according to the manufacturer’s instructions. Viral detection was conducted using a multiplex real-time PCR assay as previously described [13]. Norovirus-positive stool specimens were subjected to a conventional PCR procedure, and subsequently sequenced and genotyped. In brief, after carrying out two sets of conventional PCR using primers G1-SKF and G1-SKR to amplify the N/S domain of the norovirus capsid protein [14], the amplified products of a 387-bp fragment were sequenced using an ABI Prism 3130 Genetic Analyzer (Applied Biosystems, USA). Genotyping was performed using a public norovirus genotyping tool [15]. A phylogenetic tree was constructed using the neighbor-joining method in the MEGA program (version 6.0).

### Statistical analysis

A database was constructed using EpiData 3.1, and the statistical analysis was performed using SAS (version 9·2; SAS Institute Inc., USA). Differences between pairs of groups were analyzed using *x*^2^ tests. Univariable logistic regression was used to quantify associations between exposure and illness, and the results are presented as odds ratios (ORs) and 95% confidence intervals (CIs). All the tests were two-sided tests and a level of significance of 5% was used.

## Results

### Epidemiological investigations

A total of 19 suspect cases aged from 8 to 9 years met the criteria for AGE. They were members of 2 classes, which were designated class A and B. Class A involved grade 2 students and was on the second floor and class B involved grade 3 students and was on the third floor. The attack rates were significantly different between the two classes, at 38% in class A (15/40) and 10% in class B (4/41) (Fisher’s exact test *x*^2^ = 8.680, *P* = 0.003). Of the 19 suspect cases, seven were males (37%). Twelve cases (63%) were aged 8 years and seven (37%) were aged 9 years. For class A, the male attack rate was 32% (6/19) and the female attack rate was 43% (9/21), with no significant difference (Pearson *x*^2^ = 0.541, *P* = 0.462). All 19 cases had mild symptoms without hospitalization. They all experienced vomiting, but only three (16%) had diarrhea. Abdominal pain was reported by five (26%) cases and fever was reported by one (5%) case. The medium reported symptom duration was 2 days, with a range of 1 to 3 days (Table 1).

**Table 1:** Demographics, symptoms of the 19 cases and epidemiological parameters included in the study

Variables	Descriptions
Gender *n* (%)	
Female	12(63)
Male	7(37)
Age (years old) *n* (%)	
8	12(63)
9	7(37)
Vomiting *n* (%)	19(100)
Abdominal pain *n* (%)	5(26)
Diarrhea *n* (%)	3(16)
Fever *n* (%)	1(5)
Symptom duration (days)	
Median	2
Range	1–3
Epidemic duration (days)	7
Number of case in the 1st peak *n* (%)	15(79)
Number of case in the 2nd peak *n* (%)	4(21)
Retrospective investigation	
Contact to suspect case *(N)*	31
AGE^a^ onset *n* (%)	15(48)
Non-AGE onset *n* (%)	16(52)
Non-contact to suspect case *(N)*	28
AGE onset *n* (%)	4(14)
Non-AGE onset *n* (%)	24(86)

^a^AGE: acute gastroenteritis

**Fig. 1:**
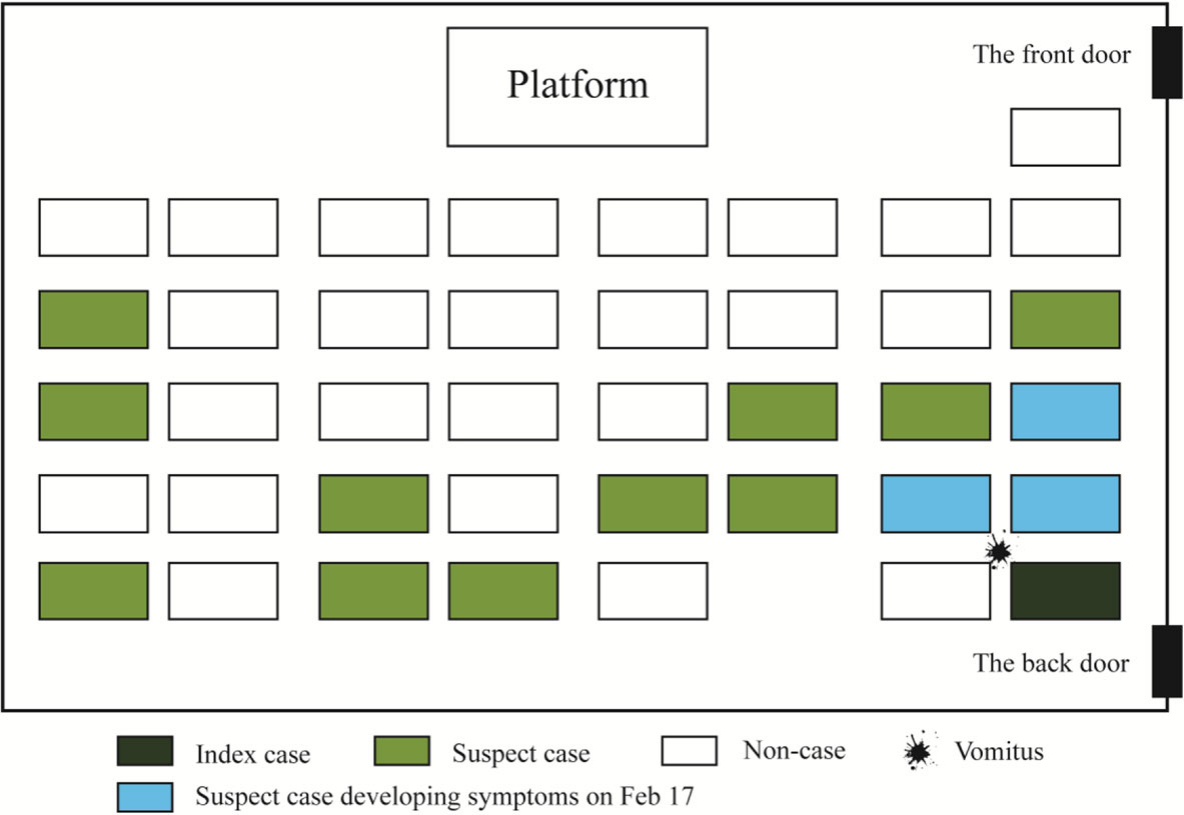
Vomitus positioning of index case and seating arrangements of students in class A of the primary school, Jinshan district, Shanghai on February 16, 2017

The first case, an 8-year-old girl in class A, had abdominal discomfort at 9 a.m. on February 16, 2017 and experienced vomiting while sitting in her seat. The vomitus was cleaned up and the floor was mopped by the teacher, and the girl continued attending class. Figure 1 shows the seating arrangements of the students in class A. The girl skipped rope with her classmates in their physical education (PE) class in the afternoon. The girl did not go to hospital for treatment and was not given any treatment by her guardian after school. As her symptoms were spontaneously alleviated at 8 p.m. the same day, the index case attended class normally the next day. Through interview, we found that the index case had amused herself by taking part in recreational activities in a crowded plaza, where she had come into close contact with other children for nearly 2 h on February 14. Other students in class A had not visited this plaza on the same day. The majority of cases occurred on February 18 (9 cases), which represented the peak of the first wave, and the last cases were reported on February 22 (three cases). A boy in class A began to vomit on February 18, and his older sister in class B began to vomit on February 20. This girl, who was the first case in class B, failed to rest at home after symptom onset, which initiated the second wave of the epidemic and led a further three students in class B to become ill 2 days later. No other cases were identified during the investigation period and the whole epidemic lasted for 7 days. Thus, the possibility of the AGE outbreak source being school lunch or drinking water contamination can be ruled out.

**Fig. 2:**
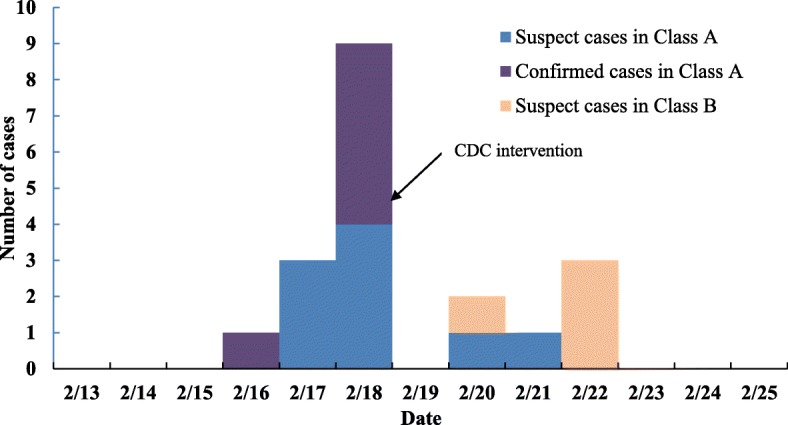
Epidemic curve of the AGE outbreak by date of onset in a primary school in Jinshan district, Shanghai, in February 2017

According to the epidemic curve (Fig. 2), the outbreak appeared to be a small-scale propagated epidemic and person-to-person transmission was the main mode of transmission. The outbreak comprised two successive peaks, with the main peak involving 15 cases in class A and the subsequent minor peak involving four cases in class B. To confirm whether the outbreak could be attributable to person-to-person transmission, we retrospectively investigated the 19 suspect cases and 40 unmatched healthy controls from the two classes (who had no measurable symptoms). They were split into two groups according to whether they had contacted a suspect case in the 3 days prior to onset (for the suspect cases) or recruitment (for the healthy controls). The results showed that the attack rate in those who contacted a suspect case was 48% (15/31) and it was 14% (4/28) in those who did not contact a suspect case, with the difference being significant (Pearson *x*^2^ = 7.836, *P* = 0.005). The unadjusted OR was 5.6 (95% CI: 1.6–20.1). It is believed that the first case in class A might have been the source and disseminator of this AGE outbreak, and the first case in class B (who was infected by her younger brother) was responsible for the second wave of the epidemic, which occurred in class B. All cases were isolated at home until 72 h after recovery from illness and other students in all classes were followed up until February 25, 2017 to see if they developed AGE symptoms. No new cases were observed during the period. This norovirus-associated AGE outbreak was then considered to have ended.

### Environmental investigations

The school has its own canteen with 10 food handlers, where lunches are prepared and served to all students and staff all year round. The hygiene inspection of the canteen identified no concerns regarding food handling, and food processing conformed to food safety management policies. None of the food handlers reported any illness involving vomiting, diarrhea, or fever in the previous month. Nevertheless, reminders to observe hygienic handwashing practices were provided to the food handlers. Purified water dispensers with automatic heating function are present on each floor of the 4-floor teaching building. Teachers and students can drink cooled or boiled water using their own bottles. The scheduled testing reports showed that the water quality met the requirements. The classroom windows were opened each morning for ventilation, and the lavatories were cleaned and disinfected with chlorine-containing disinfectants after class by cleaners.

### Microbiological investigations

Specimens were collected from six student cases (stool sample) and three canteen staff members (rectal swabs) and five environmental swabs were also collected for laboratory testing, including testing for pathogenic bacteria, rotavirus, adenovirus, astrovirus, and norovirus. They were all negative for pathogens except for genogroup I norovirus, which was found in all six stool specimens. The negative results for specimens collected from the food handlers and the canteen environmental swabs further suggested that the outbreak was unlikely to be caused by inadequate food handling practices.

**Fig. 3:**
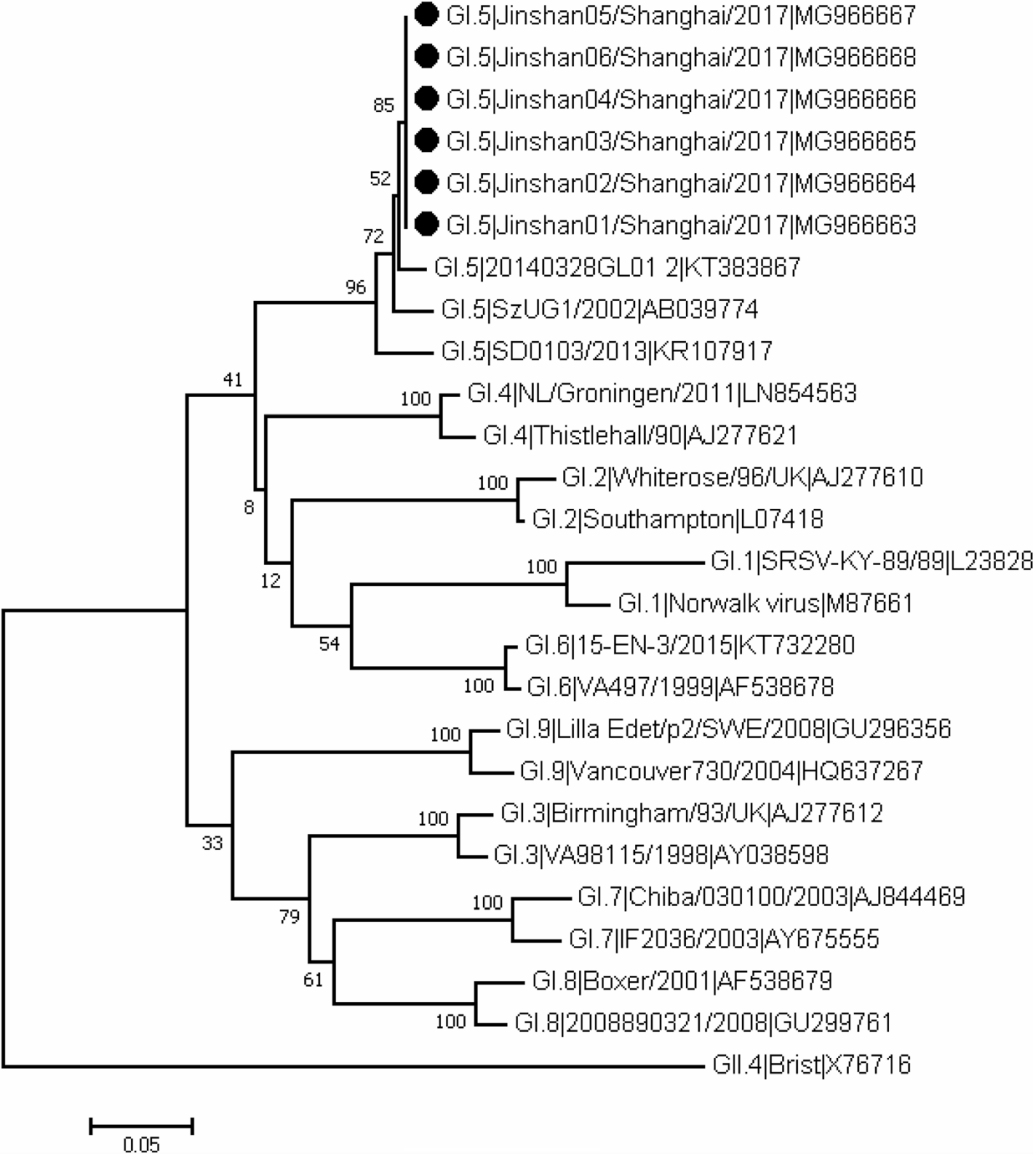
Phylogenetic tree derived from 6 GI.5 norovirus partial nucleotide sequences of capsid gene from 6 specimens (accession number:MG966663-MG966668)collected from symptomatic students. The tree was constructed by MEGA6.0. Scale bar represents numbers of substitutions per site and bootstrap values are indicated for the corresponding nodes(1000 replicates). Specimens from cases are marked by black circles

All of the six norovirus-positive specimens were genotyped, and genotype characterization identified all six isolates as GI.5, with 100% nucleotide sequence homology (Fig. 3). This implied that the GI.5 norovirus strain was the most probable cause of the AGE cases and all the cases in the school shared the same infectious source.

## Discussion

Noroviruses can cause not only sporadic gastroenteritis but also AGE outbreaks in individuals of all ages. Norovirus-associated AGE usually results in a mild self-limiting illness. However, the consequences of norovirus infection in immunodeficient individuals, the elderly, and children can be especially severe and have been previously reported to include hospitalization and death. Norovirus is associated with 18% of cases of diarrheal disease worldwide [16] and causes 70,000–200,000 deaths every year globally [17]. Although vaccines against norovirus are now under development, the genetic diversity of noroviruses has complicated this process.

It is known that human gastroenteritis is caused primarily by GI and GII noroviruses, and GII.4 noroviruses have been responsible for most AGE outbreaks in the past decade [18]. However, a newly emergent variant of the previously rare GII.17 genotype was reported to be the predominant cause of norovirus-associated AGE outbreaks during the 2014–2015 winter in some parts of Asia and it spread rapidly across the continent [19, 20]. This indicates that non-GII.4 norovirus might become the main genotype. In October–December 2016, the number of norovirus-associated AGE outbreaks in China rose steeply compared to in the same period in the previous 4 years. A recombinant GII.P16-GII.2 strain emerged after 2016 predominated in these outbreaks, accounting for 79% (44) of the 56 outbreaks [21]. Our investigation revealed that the AGE outbreak that occurred in February 2017 was caused by GI.5 norovirus, which suggested that AGE outbreaks during the peak season may be caused by different norovirus genotypes. Dábilla et al. first reported the detection of one strain of GI.5 norovirus out of 54 norovirus-positive samples from hospitalized children in Brazil in 2017 [22]. A systematic review showed that GII.4 norovirus was the most prevalent GII genotype, accounting for 65% of cases of acute sporadic gastroenteritis in children (aged ≤17 years) in sub-Saharan Africa from 1993 to 2015. GI.7 (33%) followed by GI.3 (21%) and GI.5 (17%) were the most common GI genotypes [23]. The GI.5 norovirus was first detected in AGE outpatients in China in 2011 [24]. An investigation was conducted among 4123 pediatric AGE outpatients from 2008 to 2009 in four cities in China, which found that 1067 (26%) were norovirus positive, and subsequent genotyping of 451 strains showed that 445 strains were GII.4 and only 2 strains were GI.5 [25]. AGE outbreaks caused by GI norovirus, especially solely by the GI.5 genotype, have been relatively scarce around the world. A study in Victoria, Australia, showed that AGE outbreaks caused by norovirus involved a great diversity of genotypes in 2014 to 2015, during which GII.4 was the most predominant genotype detected and only 1 out of the 287 outbreaks was caused by GI.5 [2]. Epidemiological surveillance based on a comprehensive network to cover sporadic, person-to-person outbreaks, food-borne outbreaks and water-borne outbreaks is preferable [26].

Our investigation concluded that the norovirus-associated AGE outbreak in a primary school was associated with person-to-person transmission. The phylogenetic tree that we constructed indicated that the six viral isolates were all GI.5, with 100% nucleotide sequence homology, implying that GI.5 norovirus was the most probable etiological agent of the outbreak and all cases shared the same infectious source. This is the first reported norovirus outbreak caused by GI.5 in China. Consistent with other investigations that reported person-to-person spread in AGE outbreaks involving norovirus [27, 28], our investigation further illustrates that person-to-person transmission is an important cause of norovirus-associated AGE outbreaks. Levels of norovirus AGE tend to peak during cold winters, exhibiting strong winter seasonality. The outbreak investigated in this study occurred in February, the coldest month of year in Shanghai, and thus the virus presented characteristics of winter seasonality, in line with other research [27]. Schools are the most frequent setting of norovirus outbreaks involving person-to-person transmission [27], which might be partly explained by the close contact and poor hand hygiene practice among students. The outbreak investigated in this study appeared to be a small-scale propagated epidemic, in which the first epidemic peak occurred in class A, with a primary attack rate of 38%, and the second epidemic occurred in class B, with a secondary attack rate of 10%. Zhang et al. reported an AGE outbreak in China in June 2017 with an attack rate of 53%, which is higher than those in this study [11]. A sequence analysis confirmed that GII.P16-GII.2 norovirus, the main genotype in 2016–2017 in China, was the etiological agent of the outbreak [11]. The relatively low attack rate and mild symptoms of cases in the present study implies that the virulence of the GI.5 genotype may be lower than the virulence of predominant norovirus genotypes, but further research is needed to confirm this.

Exposure to vomitus or fomites from cases contributes to person-to-person transmission of norovirus [29, 30]. According to our investigation, cases in class A were commonly exposed to the index case and the transmission mode may have included close contact in the classroom or in the PE class and exposure to vomitus. The chairs of seven of the suspect cases were around the index case, which indicated they had more exposure opportunity to the vomitus of the index case. Additionally, rope skipping in the PE class increased the possibility of person-to-person transmission of the norovirus. After a boy in class A became ill, he infected his older sister through close daily-life contact, which then led to the secondary epidemic in class B. Classrooms represent a major setting involving comparatively confined indoor environments. Our quantitative analysis indicated that contact between students and suspect cases led to a 5.6-fold increased risk of infection among these students compared with those without contact with the suspect cases. Thus, the conclusion of our epidemiological investigation was that person-to-person transmission was the most likely transmission mode in this outbreak. Considering that the index case contacted closely with some children in a crowded plaza for about 2 h, we conjectured that this episode may be linked to norovirus exposure for the index case. So it can be assumed that the index case may have been exposed to GI.5 norovirus in that plaza on February 14, which resulted in the subsequent outbreak in the school. This highlights the importance of strengthening comprehensive surveillance to cover sporadic infections and outbreaks, timely isolation of individuals who are ill, adequate hand hygiene, and proper environmental disinfection.

### Limitations

Our investigation has several potential limitations. Firstly, no samples of vomitus were collected, so we could not detect the presence of norovirus in vomitus, though access to these samples might have contributed to a fuller explanation of the transmission mode. Secondly, the cases that we investigated all involved mild symptoms and we failed to identify the asymptomatic cases as it was not possible to identify them during the AGE outbreak. Furthermore, there was a 2-day delay between illness onset and epidemiological investigation. If the surveillance system for AGE in institutional settings was more sensitive, the attack rate might have been lower.

## Conclusions

Based on all the data, it is tempting to speculate that this norovirus-associated AGE outbreak might have involved person-to-person transmission. Furthermore, the infectious agent was characterized as a GI.5 genotype norovirus, making this the first report of a GI.5 genotype norovirus outbreak in China. Prioritizing the sustained and sensitive surveillance of norovirus outbreaks in institutional settings would enable further appraisal of the public health implications of norovirus outbreaks and it would provide more information on the significance of the emergence of noroviruses with relatively rare genotypes. Recommendations for norovirus outbreak prevention and control primarily involve isolation of individuals who are ill, proper personal hygiene, and environmental disinfection.

## Abbreviations

AGE: Acute gastroenteritis
CI: Confidence interval
OR: Odds ratio
RT-PCR: Reverse transcription-polymerase chain reaction
